# Editorial: Influences in the progression of renal cell carcinoma

**DOI:** 10.3389/fonc.2022.1059615

**Published:** 2022-10-14

**Authors:** Mara Bacchiani, Antonio Andrea Grosso, Fabrizio Di Maida, Lorenzo Masieri, Andrea Minervini, Andrea Mari

**Affiliations:** ^1^ Department of Urology, University of Florence, Florence, Italy; ^2^ Department of Experimental and Clinical Medicine, University of Florence, Florence, Italy

**Keywords:** renal cell carcinoma, molecular diagnosis, molecular factors, progression, localized renal cancer

Renal cell carcinoma (RCC) is the most common kidney cancer and represents approximately 2-3% of all human tumors. Clear cell RCC (ccRCC) is the most common histological subtype, followed in the order by papillary (pRCC), chromophobe (chRCC) and collecting duct carcinoma (CDCK) (Tang et al.).

In 2020, there were approximately 74,000 new cases and 15,000 deaths in the United States. Over the years, an annual increase of about 3% in incidence has been shown in most of the world (1). The increasing incidence could be due to the wide use of ultrasound, computed tomography (CT) and magnetic resonance imaging (MRI) during screenings and medical evaluations, since more than 80% of kidney cancers are incidental findings (Liu et al.).

At diagnosis most of renal cancers are asymptomatic, non-palpable, still localized, that is intracapsular (T1 and T2) or locally advanced tumors (2).

Partial nephrectomy is currently recommended by the European Guidelines as the standard surgical treatment for cT1 (<7 cm) renal tumors because its oncologic outcomes are equivalent and its functional outcomes are superior comparing with those of radical nephrectomy (Huang et al.).

Partial nephrectomy has been also extended to clinical T2 renal tumors with promising oncological outcomes (3).

RCC presents a 5-year relative survival rate of 76%, although survival is highly dependent on the stage at diagnosis. Nevertheless, around 30% of patients with localized tumors, developed local recurrence or tumor progression within three to five years (4, 5).

Many studies have been led to identify prognostic factors to individualize surveillance and select high-recurrence risk patients which could benefit from adjuvant therapy.

Tumour grade and histotype are prognostic factors recommended by the EAU guidelines. For example, collecting duct carcinoma from the epithelium of the collecting ducts of Bellini is an aggressive malignant tumor and has a poorer prognosis and more limited response to immunotherapy comparing with most common histotypes (Tang et al.).

Others consolidated prognostic factors are TNM classification, presence of sarcomatoid or rhabdoid features, microvascular invasion (MVI), tumor necrosis, pelvicalyceal system invasion, renal vein invasion and renal sinus invasion, that is infiltration of tumor into the peri-renal fat (6, 7).

On the other hand, the lymphovascular invasion seems not to be significantly correlated to cancer-specific survival (CSS) (Guo et al.).

Positive surgical margin (PSM) is an important predictor of disease-free survival (DFS) in patients with advanced T classification, in fact positive margin determine 18-fold higher risk of recurrence (3), but PSM has no impact on cancer-specific survival after partial nephrectomy for T1 neoplasia because of low cancer progression rate (8). In fact the relationship between PSM and increased risk of recurrence after partial nephrectomy is still uncertain.

Moving from these aggressive pathological characteristics, an individualized risk-based approach might be useful to decide follow-up protocols and postoperative therapies after radical or partial nephrectomy.

There is poor level of evidence about the use of prognostic models in patients with localized RCC, such as Leibovich score and the University of California Integrated Staging System (UISS) score. However, these tools represent a useful guide for patients’ enrollment into adjuvant clinical trials.

In this special issue, several studies have been conducted to understand the molecular mechanisms underlying RCC and its recurrence and metastasis process to identify factors which influence the progression of the disease and to provide new molecular and therapeutic strategies for patients with RCC.

A review synthetized that main biological pathways altered in ccRCC are cell cycle, angiogenesis, hypoxia, and immune response (Petitprez et al.). Since the von Hippel–Lindau (VHL) gene has been identified in 1993, VHL gene mutation has been reported in 70% of ccRCC tumors and its hypermethylation in 15%. The VHL gene inactivation conducts to hypoxia-inducible factors (HIF1a-HIF1b) activation and to increasing angiogenesis through vascular endothelial growth factor (VEGF) signaling (Petitprez et al., Wang et al., Tong et al.).

Therefore, anti-angiogenic tyrosine kinase inhibitors (TKI) and monoclonal antibodies against VEGF have become a crucial treatment option for ccRCC patients (9).

VHL mutation might also be involved in immunoregulation by activating the effector T cells and enhancing cytokine level in ccRCC.

Many studies underline that extracellular matrix (ECM) and tumor microenvironment (TME) are crucial for tumor development, tumor progression and metastasis spread (Xu et al.).

In fact, several stromal and immune cells, such as myeloid inhibitory cells, tumor associated macrophages, mesenchymal stem cells and cancer-associated fibroblasts (CAFs), provide vascularization, drop immunitary defence, and build a matrix barrier in order to promote cancer cells’ survival.

As soon as the tumor is born, multiple CAFs begin to appear and secrete multiple pro-tumor factors which promote neo-angiogenesis, remodel the ECM, modify immunoregulation and favorite therapeutic resistance (Chen et al.).

Immunohistochemical identification of immune biomarkers, such as CD8 and PD-L1, led to introduction of immune checkpoint inhibitors, such as Pembrolizumab and Nivolumab, which block programmed cell death protein-1 (PD-1), or Ipilimumab, which is cytotoxic T-lymphocyte-associated protein 4 (CTLA-4) inhibitor.

PD-1 immune checkpoint inhibitors, as well as CTLA-4 inhibitors, proved themselves clinically safe and effective by improving objective response rates like progression-free survival (PFS) and overall survival (OS) and have become the first line standard of care in PDL1-positive metastatic disease (Wang et al.).

TME with its complexity is the target of last decade innovative therapies, such as immune checkpoint inhibitors (ICIs) and anti-angiogenic tyrosine kinase inhibitors (TKIs), but is also the origin of many potential therapeutic resistances.

For example the TME activation of angiogenic escape pathways decreased the therapeutic response to TKIs.

CD248, a specific biomarker of activated fibroblasts and pericytes residing in tumor blood vessels, is upregulated in RCC and may represent a novel prognostic and therapeutic target and the antibody-drug targeting CD248 might overcome the immunosuppressive TME and help to destroy cancer (Xu et al., Zhang et al.).

Moreover, adipose-related genes (ARGs) expression is associated with immune cell infiltration and immune microenvironment in ccRCC. The adipogenic transdifferentiation status of tumor cells is closely related to prognosis and ARGs expression may be a novel independent biomarker in the prediction of ccRCC patients’ survival (Wang et al.).

Others potential target are AKT and STAT3 signaling pathways, activated by aromatic hydrocarbon receptor (AhR) which is up-regulated by kynurenine (Kyn) produced by CAFs. Alternatively downregulate AhR might improve the antitumoral effects of Sorafenib or Sutinib(Chen et al.).

The urokinase-type plasminogen activator receptor (PLAUR) is upregulated in RCC, such as in several other cancers, and is associated with poor OS and DFS. PLAUR is involved in various malignancy-related processes, including angiogenesis, cell differentiation, proliferation and migration and its expression increases with tumor grade and stage. PLAUR also regulates several immune cells, such as CD4+ T cells and macrophages, which promote respectively RCC cell proliferation and invasion (Wang et al.).

Malignant cells are characterized by unlimited DNA replication. The minichromosome maintenance proteins (MCM2-7) are essential initiation factors in DNA replication and their expression is increased in ccRCC, especially in patients with a poor prognosis. Therefore, MCM2-7 overexpression is an important biomarker and a potential molecular target (Zhang et al.).

Between tumor suppressor mutated in ccRCC we can count Keratinicyte Differentiation Factor1 (KDF1). Its expression levels correlate negatively with tumor grade and tumor stage, and positively with patients’ survival. Overexpression of KDF1 is associated to reduction of ccRCC cells’ proliferation, migration and invasion (Zheng et al.).

Other tumor suppressors are Zrt‐ and Irt‐like proteins (ZIP) family members (SLC39A1-14), which function is to pass zinc into the cytoplasm for many biological anti-neoplastic processes. A study showed that down-regulation of SLC39A8 is involved in ccRCC progression and lower expression of SLC39A8 correlates to worse prognosis (Liu et al.).

As these factors may allow a better identification of patients who could respond to immunotherapy by outlining real immune signatures, it is of utmost importance to define appropriate algorithms to correctly define personalized treatments and, eventually, determine bioindicators for cancer monitoring during follow-up.

New molecular-based research on oncogenes, tumor suppressors, cancer associated fibroblasts are needed to identify biomarkers which influence RCC progression and prognosis, to guide the clinical management with personalized treatment strategies and to improve survival outcomes for RCC patients.

## Author contributions

MB: Writing original draft. AG: Writing original draft. FM: Supervision. LM: Conceptualization. AMi: Conceptualization. AMa: Conceptualization, Writing original draft, Supervision. All authors have made a substantial contribution to the manuscript and have read and approved the final manuscript.

## Conflict of interest

The authors declare that the research was conducted in the absence of any commercial or financial relationships that could be construed as a potential conflict of interest.

## Publisher’s note

All claims expressed in this article are solely those of the authors and do not necessarily represent those of their affiliated organizations, or those of the publisher, the editors and the reviewers. Any product that may be evaluated in this article, or claim that may be made by its manufacturer, is not guaranteed or endorsed by the publisher.

## References

[1] Padala SA Barsouk A Thandra KC Saginala K Mohammed A Vakiti A . Epidemiology of renal cell carcinoma. World J Oncol (2020) 11:3. doi: 10.14740/WJON1279 PMC7239575 32494314

[2] Brassetti A Anceschi U Bertolo R Ferriero M Tuderti G Capitanio U . Surgical quality, cancer control and functional preservation: Introducing a novel trifecta for robot-assisted partial nephrectomy. Minerva Urol Nefrol (2020) 72:1. doi: 10.23736/S0393-2249.19.03570-7 31833720

[3] Bertolo R Autorino R Simone G Derweesh I Garisto JD Minervini A . Outcomes of robot-assisted partial nephrectomy for clinical T2 renal tumors: A multicenter analysis (ROSULA collaborative group). Eur Urol (2018) 74:2. doi: 10.1016/j.eururo.2018.05.004 29784191

[4] Ljungberg B Albiges L Abu-Ghanem Y Bedke J Capitanio U Dabestani S . European association of urology guidelines on renal cell carcinoma: The 2022 update. Eur Urol (2022) 82(4):399–410. doi: 10.1016/j.eururo.2022.03.006 35346519

[5] Di Martino S De Luca G Grassi L Federici G Alfonsi R Signore M . Renal cancer: New models and approach for personalizing therapy. J Exp Clin Cancer Res (2018) 37:1. doi: 10.1186/s13046-018-0874-4 30185225 PMC6126022

[6] Klatte T Rossi SH Stewart GD . Prognostic factors and prognostic models for renal cell carcinoma: a literature review. World J Urol (2018) 36:12. doi: 10.1007/s00345-018-2309-4 29713755

[7] Anceschi U Ferriero MC Tuderti G Brassetti A Bertolo R Capitanio U . Head to head impact of margin, ischemia, complications, score versus a novel trifecta score on oncologic and functional outcomes after robotic-assisted partial nephrectomy: Results of a multicenter series. Eur Urol Focus (2021) 7(6):1391–99. doi: 10.1016/j.euf.2020.06.021 32675046

[8] Schiavina R Mari A Bianchi L Amparore D Antonelli A Artibani W . Predicting positive surgical margins in partial nephrectomy: A prospective multicentre observational study (the RECORd 2 project). Eur J Surg Oncol (2020) 46:7. doi: 10.1016/j.ejso.2020.01.022 32007380

[9] Sun M Shariat SF Cheng C Ficarra V Murai M Oudard S . Prognostic factors and predictive models in renal cell carcinoma: A contemporary review. Eur Urol (2011) 60:4. doi: 10.1016/j.eururo.2011.06.041 21741163

